# Risk factors that affect the degree of bronchopulmonary dysplasia in very preterm infants: a 5-year retrospective study

**DOI:** 10.1186/s12887-022-03273-7

**Published:** 2022-04-12

**Authors:** Tingting Yang, Qianqian Shen, Siyu Wang, Tianfang Dong, Liang Liang, Fan Xu, Youfang He, Chunlei Li, Fang Luo, Jiahong Liang, Chunhui Tang, Jinghui Yang

**Affiliations:** 1 The Affiliated Hospital of /College of Medicine, Kunming University of Science and Technology, Kunming, Yunnan China; 2 Department of Pediatrics, The First People’s Hospital of Yunnan Province, Kunming, Yunnan China; 3 College of Medicine, Dali University, Dali, Yunnan China; 4 Department of Pediatrics, The First Hospital of Kunming, Kunming, Yunnan China; 5 The First Clinical Medical College, Yunnan University of Traditional Chinese Medicine, Kunming, Yunnan China; 6 The Affiliated Hospital of Kunming University of Science and Technology, Kunming, Yunnan China; 7 Yunnan Province Clinical Center for Hematologic Disease, Kunming, Yunnan China

**Keywords:** Bronchopulmonary dysplasia, Risk factors, Very preterm infants

## Abstract

**Background:**

Bronchopulmonary dysplasia (BPD) is one of the most common adverse consequence of premature delivery and the most common chronic lung disease in infants. BPD is associated with long-term lung diseases and neurodevelopmental disorders that can persist into the adulthood. The adverse consequences caused by severe BPD are more serious. However, there were few studies on the risk factors for severe BPD.

**Methods:**

This is a retrospective study of preterm infants born less than 32-week gestational age (GA) and diagnosed with BPD.

**Results:**

A total of 250 preterm infants with a diagnosis of BPD and GA < 32 weeks were included (137 boys [54.8%] and 113 girls [45.2%]). The birth weight ranged from 700 g to 2010 g and the mean birth weight was 1318.52 g (255.45 g). The GA ranged from 25 weeks to 31 weeks and 6 days (mean, 30 weeks). The number of cases of mild, moderate and severe BPD were 39 (15.6%), 185 (74.0%) and 26 (10.4%), respectively. There were significant differences in the rate of small for gestational age (SGA), intrauterine asphyxia, pulmonary hemorrhage, neonatal respiratory distress syndrome (NRDS), circulatory failure, pulmonary hypertension, patent ductus arteriosus (PDA), pulmonary surfactant (PS), aminophylline, caffeine, glucocorticoids, tracheal intubation, diuretics, and parenteral nutrition length among the three groups (*P* < 0.05). The time of parenteral nutrition (aOR = 3.343, 95%*CI*: 2.198 ~ 5.085) and PDA (aOR =9.441, 95%*CI*: 1.186 ~ 75.128) were independent risk factors for severe BPD compared with mild BPD. PDA (aOR = 5.202, 95%*CI*: 1.803 ~ 15.010) and aminophylline (aOR = 6.179, 95%*CI*: 2.200 ~ 17.353) were independent risk factors for severe BPD, while caffeine (aOR = 0.260, 95%*CI*: 0.092 ~ 0.736) was the protective factor for severe BPD compared with moderate BPD. The time of parenteral nutrition (aOR = 2.972, 95%*CI*: 1.989 ~ 4.440) and caffeine (aOR = 4.525, 95%*CI*: 1.042 ~ 19.649) were independent risk factors for moderate BPD compared with mild BPD. Caffeine (aOR = 3.850, 95%*CI*: 1.358 ~ 10.916) was the independent risk factor for moderate BPD, while PDA (aOR = 0.192, 95%*CI*: 0.067 ~ 0.555) and aminophylline (aOR = 0.162, 95%*CI*: 0.058 ~ 0.455) were protective factors for moderate BPD compared with severe BPD. The time of parenteral nutrition (aOR = 0.337, 95%*CI*: 0.225 ~ 0.503) and caffeine (aOR = 0.221, 95%*CI*: 0.051 ~ 0.960) were protective factors for mild BPD compared with moderate BPD. The time of parenteral nutrition (aOR = 0.299, 95%*CI*: 0.197 ~ 0.455) and PDA (aOR = 0.106, 95%*CI*: 0.013 ~ 0.843) were protective factors for mild BPD compared with severe BPD.

**Conclusion:**

The time of parenteral nutrition is the risk factor of moderate and severe BPD. PDA and aminophylline are risk factors for severe BPD. The role of caffeine in the severity of BPD is uncertain, and SGA is not related to the severity of BPD. Severe or moderate BPD can be avoided by shortening duration of parenteral nutrition, early treatment of PDA, reducing use of aminophylline and rational use of caffeine.

**Trial registration:**

Retrospectively registered.

## Background

BPD is a chronic lung disease. Northway [1] reported BPD for the first time in 1967. However, the definition and characteristics of BPD have changed over the past several decades [1, 2]. The new definition of BPD is more comprehensive, with oxygen support at least 28 days as required, and includes a classification of severity. Infants born at GA < 32 weeks were classified as mild (no need for oxygen), moderate (21 to 30%) or severe BPD (> 30% or positive pressure assistance) based on the required fraction of inspired oxygen (FiO_2_) at 36 weeks of corrected GA [2].

In recent few decades, due to the development of perinatology and neonatal care, such as prenatal corticosteroids and exogenous PS treatment, the survival rate of premature infants has dramatically increased. The incidence of many complications related to premature delivery, such as NRDS, ventricular hemorrhage and necrotizing enterocolitis (NEC), has decreased. However, BPD remains one of the most common complications in preterm infants [3, 4]. Moreover, the incidence rate of BPD is becoming higher and higher, probably due to the increasing survival rate of very preterm infants [5]. According to the studies of different neonatal networks in many countries including the United States, Canada, South Korea, China and India, the incidence rate of BPD varies greatly, ranging from 11 to 50%, because of different diagnostic and management criteria [6]. The incidence rate of BPD increases with the decrease of GA or birth weight. About 30% of very low birth weight premature infants suffer from BPD [7]. The previous studies has reported that the incidence rate of BPD in preterm infants with GA < 32 weeks fluctuated from 12.9 to 41% [8].About 40 ~ 50% of extremely premature infants suffer from BPD [4, 9], and the incidence rate of preterm infants with GA < 25 weeks is as high as 80% [10].

Newborns suffering from BPD have higher mortality [11] and incidence of pulmonary, cardiovascular, and neurodevelopmental disorders, leading to lower quality of life and increased resource utilization [12, 13]. Premature infants with severe BPD are particularly difficult to manage and are more prone to a variety of complications and comorbidities, including prolonged hospitalization, requirement of family respiratory support and higher death risk [14, 15]. The mortality of premature infants with severe BPD is 25%, and the readmission rate of surviving premature infants in the first year is as high as 50%. The incidence of neurodevelopmental disorder is 2-3 times higher than that of normal infants, which has become one of the important factors affecting the quality of life of surviving premature infants. Therefore, it’s particularly important to avoid severe BPD.

In the past few decades, many clinical prediction models have been reported. As we know, the smaller GA or birth weight, the higher the incidence of BPD. Other perinatal factors affecting BPD include gender [16], intrauterine growth retardation [17], chorioamnionitis [18], smoking [19], and race/nation [17]. Some postnatal factors also raise the risk of BPD and adverse outcomes such as NRDS, pulmonary inflammation, pulmonary vascular disease, infection, PDA, undernutrition and the need for invasive mechanical ventilation [20]. However, most studies focused on the pathogenesis and clinical risk factors of BPD, and there are few studies on the risk factors that affect the occurrence of different degrees of BPD. It is still not clear whether the above factors would increase the incidence of moderate or severe BPD. Early detection of risk factors may avoid the occurrence of moderate or severe BPD in the near future and reduce its long-term negative effects. Therefore, we put forward a hypothesis: the risk factors affecting the severity of BPD can be obtained by comparing the prenatal and postnatal conditions of preterm infants with mild, moderate and severe BPD, so as to guide clinical treatment and avoid moderate or severe BPD.

This study analyzed the sociodemographic data, perinatal factors, treatment and complications of premature infants with different degrees of BPD to find out the risk factors affecting the severity of BPD.

## Methods

### Study design and participants

This is a retrospective study. All clinical data of premature infants with GA < 32 weeks admitted to the First People’ s Hospital of Yunnan Province from January 1, 2016 to December 31, 2020 were collected. Preterm infants with severe malformations, chromosomal abnormalities, genetic metabolic diseases or incomplete information were excluded. Premature infants with GA < 32 weeks who were diagnosed with BPD were included and divided into three groups according to the severity of BPD.

### Study factors

We selected the possible risk factor variables according to previous studies. All methods were in accordance with relevant guidelines and provisions. The following data were collected:

1. Sociodemographic variables and perinatal factors: gender, GA, birth weight, intrauterine asphyxia, amniotic fluid pollution, placental abruption, premature rupture of membranes (PROM), maternal age, prenatal use of glucocorticoids, gestational diabetes mellitus (GDM), hypertensive disorder complicating pregnancy (HDCP), hyperthyroidism, hypothyroidism, maternal anemia, prenatal fever.
2. Complications: septicemia, sepsis, pneumonia, infection, NEC, apnea, respiratory failure, pulmonary hemorrhage, acute respiratory distress syndrome (ARDS), NRDS, circulatory failure, anemia, pulmonary hypertension, PDA, hypoproteinemia, electrolyte disorder, retinopathy of prematurity (ROP), intraventricular hemorrhage, intracranial hemorrhage, and paraventricular leukomalacia.
3. Treatment: PS, aminophylline, caffeine, glucocorticoid hormones, diuretics, noninvasive assisted ventilation, tracheal intubation, parenteral nutrition, FiO_2._

### Diagnostic criteria

1. Pulmonary hypertension: Pulmonary hypertension was diagnosed by echocardiograms and all newborns have echocardiograms within the first week after birth. Pulmonary artery systolic pressure higher than 35 mmHg was diagnosed as pulmonary hypertension.
2. Asphyxia: 1) The umbilical cord blood pH < 7.15; 2) Apgar score at 1 min or 5 min was less than 7 points. If the 5-min Apgar score was less than 7, the resuscitation measures were continued for 20 min, meanwhile, the Apgar scores were measured every 5 min; 3) Indications of neonatal neurological symptoms, such as seizures, coma, or hypotonia; and 4) Excluding asphyxiation from other causes.
3. PDA: The diagnosis mainly depends on echocardiography. Preterm infants have echocardiographic examinations within the first week after birth.

### Ethics

This study was approved by the Ethics committee of The First People’s Hospital of Yunnan Province (Ethic number: KHLL2021-KY001). Informed consent was waived due to the retrospective nature of this study.

### Statistical analysis

SPSS 26.0 software was used for statistical analysis. The Chi-square test or Fisher exact test was used to analyze categorical variables. Continuous data with a normal distribution was described as mean and standard deviation and was analyzed by one-way analysis of variance. Continuous data that did not conform to a normal distribution was presented as the median [25th percentile, 75th percentile] and compared among groups by Kruskal-Wallis H test. Multivariate regression model was used for statistically significant variables in single factor stepwise analysis process. The final selected factors were used as hybrid factors to calculate the adjusted advantage ratio, the maximum likelihood ratio and the corresponding 95% confidence interval. All hypothesis tests were double-tailed, *P* < 0.05 was statistically significant. G*Power (version: 3.1.9.7) was used to calculate sample size and power value.

## Results

**Fig. 1:**
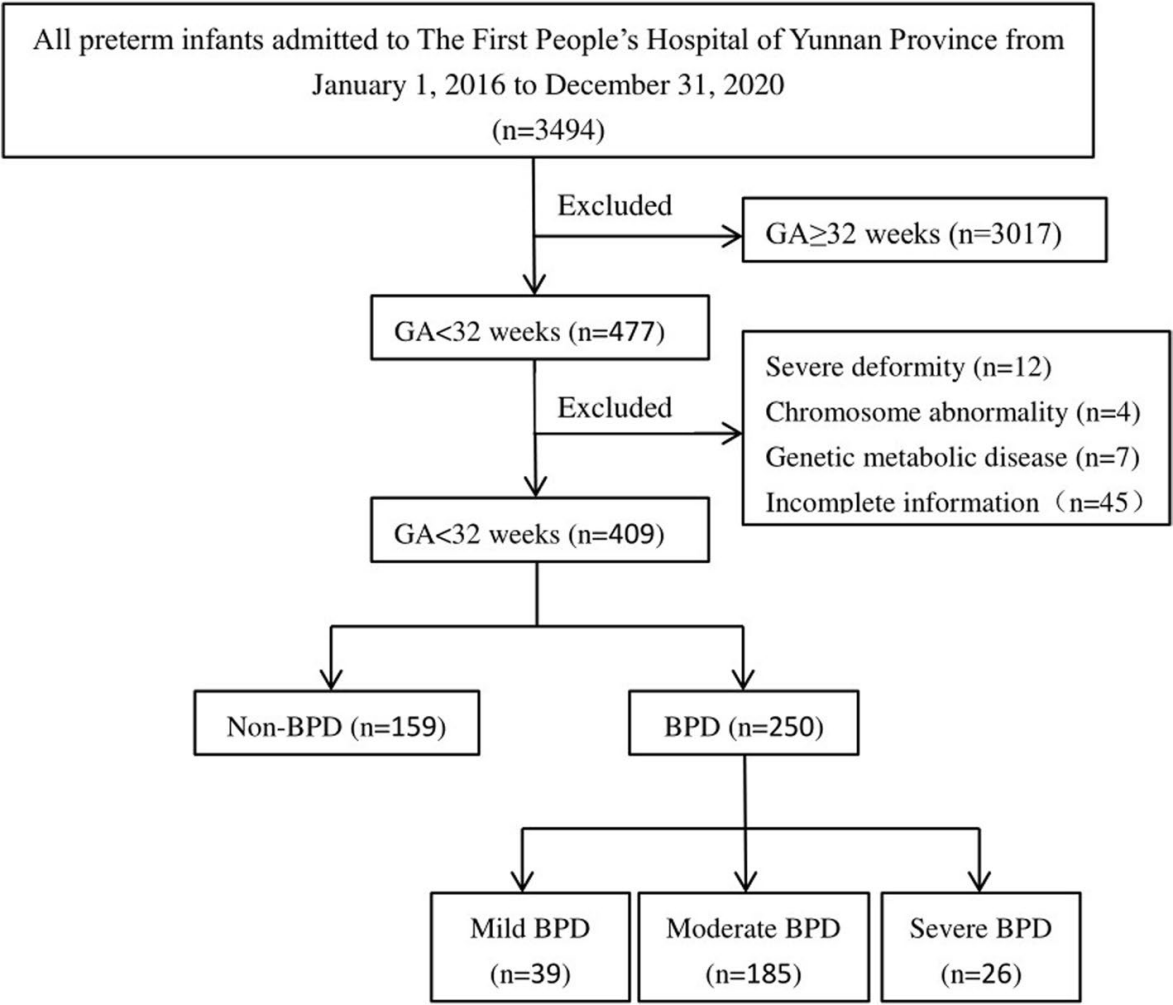
Flow chart in selection of preterm infants with BPD

All clinical data of 3494 premature infants admitted to the First People’s Hospital of Yunnan Province from January 1, 2016 to December 31, 2020 were collected, including 477 premature infants with GA < 32 weeks. As a result, 250 premature infants with GA < 32 weeks diagnosed with BPD were included (Fig. 1). Among the 250 very preterm infants with BPD, 137(54.8%) were male and 113(45.2%) were female. The average birth weight was 1318.52 g (ranging from 700 g to 2010 g). The median of GA was 30 weeks (ranging from 25 weeks to 31^+ 6^ weeks). Among 250 cases of very preterm infants diagnosed with BPD, there were 23(9.2%) with GA < 28 weeks, 96 (38.4%) with 28 ~ 29^+ 6^weeks, and 131(52.4%) with 30 ~ 31^+ 6^ weeks.

The antenatal features and demographic of very premature infants with different degrees of BPD were summarized (Table 1). The significant differences were observed in the rates of SGA and intrauterine asphyxia among the three groups (*P* < 0.05). There were no significant differences in the rates of male, GA, placental abruption, PROM, amniotic fluid pollution, maternal age ≥ 35 years, HDCP, GDM, hypothyroidism, hyperthyroidism, anemia, prenatal fever and prenatal use of glucocorticoids.

**Table 1:** Demographic features of preterm infants with different levels of BPD

Variables	Mild BPD (*n* = 39)	Moderate BPD (*n* = 185)	Severe BPD (*n* = 26)	χ^2^/H/F	*P*
Male (n, %)	23 (58.9)	98 (52.9)	16 (61.5)	1.000	0.606^#^
GA [weeks, M(P25,P75)]^a^	30.1 (29.7,31.0)	30.0 (28.8,30.8)	29.9 (28.4,31.0)	2.291^c^	0.318
SGA (n, %)	1 (2.6)	15 (8.1)	5 (19.2)	5.218	0.022*
Birth weight (g, mean ± S)^b^	1471.5 ± 213.5	1308.7 ± 246.3	1158.8 ± 265.5	13.431^d^	< 0.001
Asphyxia (n, %)	13 (33.3)	87 (47.0)	19 (73.0)	9.973	0.007^#^
Placental abruption (n, %)	0 (0)	4 (2.1)	0 (0)	0.043	0.837*
PROM (n, %)	17 (43.5)	82 (44.3)	13 (50)	0.324	0.850^#^
Amniotic fluid contamination (n, %)	0 (0)	1 (0.5)	1 (3.8)	2.378	0.123*
Maternal Age ≥ 35 years (n, %)	9 (23.0)	45 (24.3)	10 (38.4)	2.547	0.280^#^
HDCP (n, %)	6 (15.3)	40 (21.6)	7 (26.9)	0.011	0.918*
GDM (n, %)	9 (23.0)	50 (27.0)	5 (19.2)	0.882	0.643^#^
Hyperthyroidism (n, %)	0 (0)	4 (2.1)	0 (0)	0.043	0.837*
Hypothyroidism (n, %)	2 (5.1)	12 (6.4)	5 (19.2)	4.356	0.113*
Anemia (n, %)	3 (7.6)	6 (3.2)	1 (3.8)	1.396	0.498*
Antenatal fever (n, %)	1 (2.5)	6 (3.2)	0 (0)	0.230	0.631*
Use of glucocorticoids (n, %)	3 (7.6)	11 (5.9)	2 (7.6)	0.236	0.889*

*GA* Gestational age, *GDM* Gestational diabetes mellitus, *HDCP* Hypertensive disorder complicating pregnancy, *PROM* Premature rupture of membranes, *SGA* Small for gestational age

*Fisher’s exact probability method

^#^Pearson’s chi-square value

^a^Kruskal-Wallis test

^b^One-way ANOVA, the rest using chi-square test

^c^H value

^d^F value, the rest are χ^2^ values

The complications of very premature infants with different degrees of BPD were summarized (Table 2). The differences of the rates of pulmonary hemorrhage, NRDS, circulatory failure, pulmonary hypertension and PDA were statistically significant among the three groups (*P* < 0.05). And the rates of septicemia, sepsis, pneumonia, infection, NEC, apnea, respiratory failure, ARDS, anemia, hypoproteinemia, electrolyte disturbance, ROP, intraventricular hemorrhage, intracranial hemorrhage, and paraventricular leukomalacia were not significant different.

**Table 2:** Complications of preterm infants with different levels of BPD

Variables	Mild BPD (*n* = 39)	Moderate BPD (*n* = 185)	Severe BPD (*n* = 26)	χ^2^	*P*
Septicemia (n, %)	0 (0)	3 (1.6)	1 (3.8)	1.435	0.231
Sepsis (n, %)	2 (5.1)	28 (15.1)	6 (23.0)	4.944	0.084*
NEC (n, %)	1 (2.5)	6 (3.2)	1 (3.8)	0.087	0.769*
Infection (n, %)	18 (46.1)	115 (62.1)	14 (53.8)	3.701	0.157
Pneumonia (n, %)	21 (53.8)	98 (52.9)	10 (38.4)	2.016	0.365
Apnea (n, %)	4 (10.2)	38 (20.5)	7 (26.9)	3.149	0.207
Respiratory failure (n, %)	0 (0)	7 (3.7)	2 (7.6)	2.718	0.099
Pulmonary hemorrhage (n, %)	1 (2.5)	19 (10.2)	10 (38.4)	17.052	< 0.001*
NRDS (n, %)	7 (17.9)	81 (43.7)	19 (73.0)	19.647	< 0.001
ARDS (n, %)	0 (0)	2 (1.0)	0 (0)	0.021	0.884*
Circulatory Failure (n, %)	0 (0)	28 (15.1)	6 (23.0)	13,460	0.001*
Pulmonary hypertension (n, %)	0 (0)	0 (0)	1 (3.8)	4.301	0.038*
Anemia (n, %)	35 (89.7)	165 (89.1)	26 (100.0)	5.569	0.062*
PDA (n, %)	5 (12.8)	53 (28.6)	12 (46.1)	8.746	0.013
Hypoproteinemia (n, %)	21 (53.8)	116 (62.7)	20 (76.9)	3.566	0.168
Electrolyte disorders (n, %)	17 (43.5)	86 (46.4)	17 (65.3)	3.622	0.164
ROP (n, %)	6 (15.3)	40 (21.6)	4 (15.3)	1.169	0.557
Paraventricular white matter softening (n, %)	1 (2.5)	3 (1.6)	0 (0)	0.617	0.432*
Intraventricular hemorrhage (n, %)	0 (0)	1 (0.5)	0 (0)	0.011	0.918*
Intracranial hemorrhage (n, %)	7 (17.9)	44 (23.7)	7 (26.9)	0.841	0.657

*ARDS* Acute Respiratory Distress Syndrome, *NEC* Necrotizing Enterocolitis, *NRDS* Neonatal Respiratory Distress Syndrome, *PDA* Patent ductus arteriosus, *ROP* Retinopathy of Prematurity

*Fisher’s exact probability method, the rest are Pearson’s chi-square values

Treatment were also compared (Table 3). The use of PS, aminophylline, caffeine, glucocorticoids, tracheal intubation, diuretics, and parenteral nutrition were statistically significant among the three groups (*P* < 0.05). There were no significant differences in the maximum FiO_2_ ≥ 40% or noninvasive assisted ventilation.

**Table 3:** Treatment in preterm infants with different levels of BPD

Variables	Mild BPD (*n* = 39)	Moderate BPD (*n* = 185)	Severe BPD (*n* = 26)	χ^2^/H	*P*
PS (n, %)	6 (15.3)	85 (45.9)	19 (73.0)	22.166	< 0.001^#^
Aminophylline (n, %)	4 (10.2)	20 (10.8)	11 (42.3)	14.522	0.001*
Caffeine (n, %)	15 (0.38)	128 (69.1)	11 (42.3)	17.423	< 0.001^#^
Glucocorticoids (n, %)	37 (94.8)	140 (75.6)	26 (100.0)	20.606	< 0.001*
Non-invasive respiratory support (n, %)	37 (94.8)	180 (97.2)	26 (100.0)	1.532	0.217*
Invasive respiratory support (n, %)	10 (25.6)	111 (60.0)	21 (80.7)	22.291	< 0.001^#^
Maximum FiO2 ≥ 40% (n, %)	24 (61.5)	131 (70.8)	22 (84.6)	4.019	0.134^#^
Parenteral nutrition (days,M(P25,P75))^a^	10 (8,12)	17 (15,20)	19 (16,22)	82.257^b^	< 0.001
Diuretics (n, %)	19 (48.7)	50 (27.0)	15 (57.6)	14.342	0.001^#^

*FiO*_*2*_ Fraction of inspired oxygen, *PS* Pulmonary Surfactant

*Fisher’s exact probability method

^#^Pearson’s chi-square value

^a^Kruskal-Wallis test, the rest using chi-square test

^b^H value, the rest are χ^2^ values

In the univariate analysis, 14 variables were statistically significant. Then multivariate logistic regression analysis was used because the data among the three groups did not meet the parallel test. The time of parenteral nutrition and PDA were independent risk factors for severe BPD compared with mild BPD. PDA and aminophylline were independent risk factors for severe BPD, while caffeine was the protective factor for severe BPD compared with moderate BPD. The time of parenteral nutrition and caffeine were independent risk factors for moderate BPD compared with mild BPD. Caffeine was the independent risk factor for moderate BPD, while PDA and aminophylline were protective factors for moderate BPD compared with severe BPD. The time of parenteral nutrition and caffeine were protective factors for mild BPD compared with moderate BPD. The time of parenteral nutrition and PDA were protective factors for mild BPD compared with severe BPD (Table 4).

**Table 4:** Correlative factors of BPD with different degrees

	β	SE	Wald	aOR	95%*CI*	*P*
**Correlative factors for mild BPD**						
Compared with moderate BPD group						
PN (days)	−1.089	0.205	28.281	0.337	0.225 ~ 0.503	< 0.001
Caffeine	−1.510	0.749	4.059	0.221	0.051 ~ 0.960	0.044
Compared with severe BPD group						
PN (days)	−1.207	0.214	31.823	0.299	0.197 ~ 0.455	< 0.001
PDA	−2.245	1.058	4.501	0.106	0.013 ~ 0.843	0.034
**Correlative factors for moderate BPD**						
Compared with mild BPD group						
PN (days)	1.089	0.205	28.281	2.972	1.989 ~ 4.440	< 0.001
Caffeine	1.510	0.749	4.059	4.525	1.042 ~ 19.649	0.044
Compared with severe BPD group						
PDA	−1.649	0.541	9.305	0.192	0.067 ~ 0.555	0.002
Caffeine	1.348	0.532	6.428	3.850	1.358 ~ 10.916	0.011
Aminophylline	−1.821	0.527	11.948	0.162	0.058 ~ 0.455	0.001
**Correlative factors for severe BPD**						
Compared with mild BPD group						
PN (days)	1.207	0.214	31.823	3.343	2.198 ~ 5.085	< 0.001
PDA	2.245	1.058	4.501	9.441	1.186 ~ 75.128	0.034
Compared with moderate BPD group						
PDA	1.649	0.541	9.305	5.202	1.803 ~ 15.010	0.002
Caffeine	−1.348	0.532	6.428	0.260	0.092 ~ 0.736	0.011
Aminophylline	1.821	0.527	11.948	6.179	2.200 ~ 17.353	0.001

Only statistically significant items are listed

*BPD* Bronchopulmonary dysplasia, *PDA* Patent ductus arteriosus, *PN* Parenteral nutrition, *aOR* Adjusted odds ratio, *95% CI* 95% confidence interval

## Discussion

We found that the time of parenteral nutrition was the risk factor of moderate and severe BPD, PDA and aminophylline were risk factors for severe BPD, the role of caffeine in the severity of BPD was uncertain, and SGA was not related to the severity of BPD.

There was no significant difference in GA among the three groups of preterm infants with different degrees of BPD, but the rate of SGA was statistically significant, suggesting that there may have high incidence of growth retardation in the moderate or severe BPD group. Some studies have shown that fetal growth retardation raised the risk of BPD in premature infants [17, 21]. Similarly, there are evidences suggesting that SGA preterm neonates were considered to have higher risk of BPD compared to appropriate for GA neonates [22]. It was reported that SGA birth was associated with severity of BPD [23] and SGA was one of the significant risk factors for moderate or severe BPD [24]. Our study results have showed that the rate of SGA was not related to the severity of BPD, which differs from the previous studies. However, it was concluded that the risk for neonatal adverse outcome associated with SGA varies by GA [24, 25]. It can explain why the rate of SGA is not related to the severity of BPD in our study because of the low enrollment of extremely premature infants.

In our study, extremely premature infants accounted for only 9.2% maybe due to lower live-birth ratio and higher post-natal mortality in extremely premature infants. A recent study by Chinese scholars have shown only 241(20.7%) survived of the 1163 extremely premature infants, while 849(73.0%) died in the delivery room and 73(6.3%) died in the neonatal intensive care unit [26]. In our another six-year retrospective study, among 3948 hospitalized premature infants, there were only 43(1.1%) cases of extremely premature infants, 3(7.0%) cases of whom died in the neonatal intensive care unit. Besides, it may be related to high rate of giving up treatment. It was reported that 862 (74.1%) of 1163 extremely premature infants died from withholding or withdrawal of care [26]. In the future, the improvement of perinatal and neonatal care can lead to a high survival rate of extremely premature infants and the correlation between GA and severity of BPD may be described more accurately. It is necessary to have a multi-center, large sample from the real-world to find the relationship between GA and the severity of BPD.

Our data showed that the time of parenteral nutrition was an independent risk factor for moderate or severe BPD. Premature infants usually cannot tolerate enteral feeding well [27], they rely on parenteral nutrition to meet the energy demand and the optimal growth and development in the first week after birth. Parenteral nutrition is a common clinical practice, which can provide some short-term development benefits for premature infants. However, parenteral nutrition is lower caloric intake, while higher fluid intake compared with enteral feeding [28]. As is known, early nutritional support may avoid severe BPD, but low caloric intake and high fluid intake are related to the severity of BPD [29]. Our finding was consistent with previous studies. Further well-designed prospective cohort studies may better demonstrate the relationship between parenteral nutrition duration and the severity of BPD.

PDA is an important risk factor for BPD [27]. However, there are few studies about PDA is associated with the severity of BPD. Our results suggested that PDA was one of risk factors for severe BPD. Similarly, some studies have shown that PDA is a significant risk factor for developing moderate or severe BPD [24]. Besides, both medical and surgical therapies for PDA are associated with severe BPD [30, 31]. In our study, all preterm infants with PDA were treated conservatively or with medication. It is necessary to carry out a multi-center and large sample research to obtain the relationship between PDA and the severity of BPD, and pay more attention to the treatments of PDA.

Our results suggested that caffeine was a protective factor that can prevent very preterm infants from developing into severe BPD, while caffeine was one of the independent risk factors for moderate BPD. The role of caffeine in the severity of BPD was uncertain. Caffeine treatment test of premature infants with apnea confirmed that caffeine significantly reduced the occurrence of BPD [32, 33], and most data [32, 34] supported that caffeine could alleviate the severity of BPD. Moreover, the preventive effect of caffeine on moderate or severe BPD was related to its dosage [35]. Infants in the high dosage group had lower odds of developing moderate or severe BPD compared with the lower dosage group [35]. The dosage of caffeine was not included in our study, which may explain why the role of caffeine in the severity of BPD was uncertain. Large prospective studies are needed to determine the relationship between caffeine and the severity of BPD, and the dosage of caffeine should be pay more attention to.

Our study has some advantages. Multiple variables were studied including detailed maternal-antenatal pathology, such as HDCP, diabetes and thyroid status. Besides, all data came from real-world. Our results suggested that the time of parenteral nutrition was one of risk factors of moderate and severe BPD, PDA and aminophylline were risk factors for severe BPD, the role of caffeine in the severity of BPD was uncertain, and SGA was not related to the severity of BPD. However, there are limitations. First, this is a retrospective study and our data may be biased. Secondly, it is a single-center study of preterm infants (GA < 32 weeks) born in Yunnan province in China and the results should be carefully applied in other regions or country. It is necessary to have a multi-center, large sample from the real-world to obtain the relationship between SGA of BPD. Further well-designed prospective cohort researches are also needed to confirm the association between caffeine, aminophylline, parenteral nutrition duration and the severity of BPD.

## Conclusions

Severe or moderate BPD can be avoided by shortening duration of parenteral nutrition, early treatment of PDA, reducing use of aminophylline and rational use of caffeine.

## Data Availability

The datasets generated and/or analyzed during the current study are not publicly available but are available from the corresponding author on reasonable request.

## Abbreviations

ARDS: Acute respiratory distress syndrome
BPD: Bronchopulmonary dysplasia
FiO_2_: Fraction of inspired oxygen
GA: Gestational age
GDM: Gestational diabetes mellitus
HDCP: Hypertensive disorder complicating pregnancy
NEC: Necrotizing enterocolitis
NRDS: Neonatal respiratory distress syndrome
PDA: Patent ductus arteriosus
PROM: Premature rupture of membranes
PS: Pulmonary surfactant
ROP: Retinopathy of prematurity
SAG: Small for gestational age
